# Resident c-kit^+^ cells in the heart are not cardiac stem cells

**DOI:** 10.1038/ncomms9701

**Published:** 2015-10-30

**Authors:** Nishat Sultana, Lu Zhang, Jianyun Yan, Jiqiu Chen, Weibin Cai, Shegufta Razzaque, Dongtak Jeong, Wei Sheng, Lei Bu, Mingjiang Xu, Guo-Ying Huang, Roger J. Hajjar, Bin Zhou, Anne Moon, Chen-Leng Cai

**Affiliations:** 1 Department of Developmental and Regenerative Biology, The Black Family Stem Cell Institute, and The Mindich Child Health and Development Institute, Icahn School of Medicine at Mount Sinai, New York, New York 10029, USA; 2 Department of Medicine, Cardiovascular Research Center, Icahn School of Medicine at Mount Sinai, New York, New York 10029, USA; 3 Cardiovascular Center, Children's Hospital of Fudan University, Shanghai 201102, China; 4 Leon H. Charney Division of Cardiology, Department of Medicine, New York University School of Medicine, New York, New York 10016, USA; 5 Department of Biochemistry and Molecular Biology, Sylvester Comprehensive Cancer Center, University of Miami Miller School of Medicine, Miami, Florida 33136, USA; 6 Department of Genetics, Albert Einstein College of Medicine of Yeshiva University, Bronx, New York 10461, USA; 7 Weis Center for Research, Geisinger Clinic, Danville, Pennsylvania 17822, USA

## Abstract

Identifying a *bona fide* population of cardiac stem cells (CSCs) is a critical step for developing cell-based therapies for heart failure patients. Previously, cardiac c-kit^+^ cells were reported to be CSCs with a potential to become myocardial, endothelial and smooth muscle cells *in vitro* and after cardiac injury. Here we provide further insights into the nature of cardiac c-kit^+^ cells. By targeting the *c-kit* locus with multiple reporter genes in mice, we find that *c-kit* expression rarely co-localizes with the expression of the cardiac progenitor and myogenic marker *Nkx2.5*, or that of the myocardial marker, cardiac *troponin T (cTnT)*. Instead, c-kit predominantly labels a cardiac endothelial cell population in developing and adult hearts. After acute cardiac injury, c-kit^+^ cells retain their endothelial identity and do not become myogenic progenitors or cardiomyocytes. Thus, our work strongly suggests that c-kit^+^ cells in the murine heart are endothelial cells and not CSCs.

## Abstract (web summary)

The issue whether the cell surface protein c-kit identifies resident cardiac stem cells (CSC) is controversial. By using novel reporter mouse models, Sultana *et al.* show that c-kit^+^ cells represent a subpopulation of endothelial cells in the developing and adult heart and do not exhibit CSC traits in health or disease.

## Graphical abstract

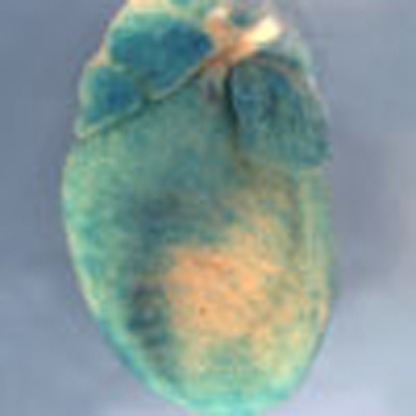

Myocardial loss or dysfunction from ischaemic heart disease is the leading cause of mortality worldwide^1^. Cardiac stem cell (CSC) transplantation is a potential therapeutic intervention for patients with heart failure^2^,^3^,^4^,^5^,^6^. For these reasons, considerable efforts have been made to identify specific markers of stem cells with cardiomyogenic potential^2^,^3^,^4^,^7^,^8^,^9^,^10^. Among the various markers employed to identify resident CSCs, c-kit has played a prominent role^11^,^12^. Cardiac c-kit-positive (c-kit^+^) cells were the first population of putative CSCs described as being negative for the blood lineage marker (Lin^−^) in the adult rat heart, along with possessing self-renewing, clonogenic and multipotent characteristics^7^,^13^,^14^. c-kit^+^ cardiac cells were also shown to be necessary and sufficient for myocardial regeneration after cardiac injury in rats and mice^15^. While these findings present encouraging therapeutic possibilities, controversy regarding their use remains due to divergent observations about the myogenic potential of resident c-kit^+^ cells in the mammalian heart^16^,^17^,^18^. A recent lineage tracing study of c-kit progeny revealed that c-kit^+^ cells have extremely limited potential to differentiate into cardiomyocytes during aging and after injury in mice^19^. However, doubts have been cast on this study, questioning not only the efficiency of Cre/LoxP recombination for lineage tracing but also potentially unreliable or insensitive expression of both the Cre drivers and reporter mouse models employed^20^,^21^. Thus, important questions remain unanswered. For example, what is the exact nature of cardiac c-kit^+^ cells? Do these cells give rise to multiple cardiac lineages during development and after heart failure? Do the benefits (if any) of c-kit^+^ cell-based therapies arise from an ability to differentiate into cardiomyocytes, or do c-kit^+^ cells generate paracrine factors or a paracellular environment that promotes recovery? Given that c-kit^+^ cells are being clinically tested on human patients with ischaemic cardiomyopathy^21^,^22^,^23^, fully addressing these questions is critical.

Determining the identity of c-kit^+^ cells in the mammalian heart is the foundation for answering these questions. To overcome the limitations of antibody-based immunostaining or transgenic mouse lines with partial regulatory elements that may not be sufficiently sensitive and/or faithfully reproduce the endogenous expression of a gene, we generated a series of mouse models that allow precise characterization of the identity of c-kit^+^ cells and their progeny in mouse hearts. Using these models, we unexpectedly found that cardiac c-kit-expressing cells are actually a subpopulation of endothelial cells in the developing and adult heart in mice. c-kit^+^ cells rarely express the cardiac progenitor marker Nkx2.5 or the differentiated cardiomyocyte marker cardiac troponin T (cTnT), nor do they become cardiomyocytes during development or after injury. Our results strongly suggest that c-kit^+^ cells in the mammalian heart are actually endothelial cells and not CSCs.

## Results

### c-kit is expressed in the developing and adult mouse heart

**Figure 1:**
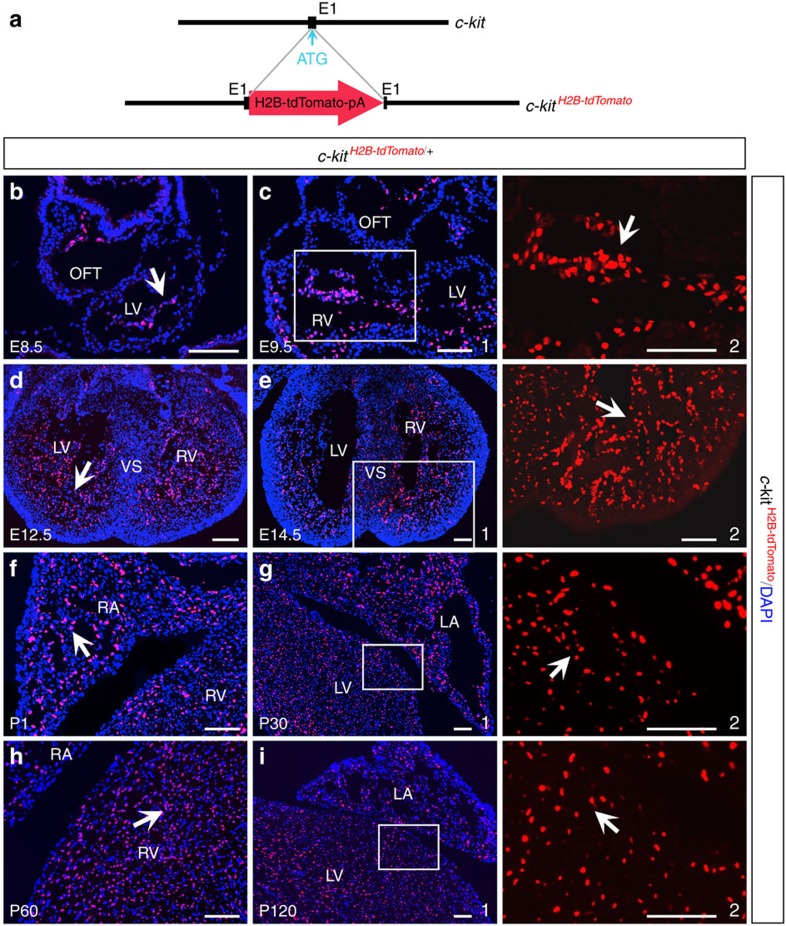
Cardiac c-kit expression in *c-kit*^*H2B-tdTomato/+*^mice. (**a**) Diagram of the *c-kit*^*H2B-tdTomato/+*^knock-in allele. (**b**–**i**) Sections of *c-kit*^*H2B-tdTomato/+*^hearts at embryonic days (E) 8.5, 9.5, 12.5 and 14.5 (**b**–**e**) and at postnatal (P) days 1, 30, 60 and 120 (**f**–**i**). c2, e2, g2 and i2 are high-magnification images (without DAPI) of the areas outlined in c1, e1, g1 and i1, respectively. c-kit^H2B-tdTomato^ cells are denoted by arrows. LA, left atria; LV, left ventricle; OFT, outflow tract; RA, right atria; RV, right ventricle; VS, ventricular septum. *n*=3 for each stage. Scale bar, 100 μm.

We first generated a knock-in mouse model, *c-kit*^*H2B-tdTomato/+*^, by gene targeting (Fig. 1a and Supplementary Fig. 1). In this animal, the *H2B-tdTomato* cassette was inserted into the *c-kit* start codon without deleting any genomic sequences, thereby expressing tdTomato under the control of the full complement of endogenous *c-kit* regulatory elements. Since *tdTomato* is fused to histone *H2B* gene^24^, its expression is localized to the nucleus.

To confirm the fidelity of the c-kit^H2B-tdTomato^ signal to the endogenous c-kit expression pattern, we performed whole-mount RNA *in situ* hybridization on the wild-type mice from embryonic day (E) 9.5 to E13.5. By comparing c-kit^H2B-tdTomato^ signals to c-kit mRNA expression, we found that the signals overlapped in all known regions of c-kit expression^25^,^26^, such as the pharyngeal arches, liver, umbilical cord and melanocytes (Supplementary Fig. 2a–c). Furthermore, H2B-tdTomato expression was detected in other organs, including the lung, stomach, intestine and spleen (Supplementary Fig. 2e), as well as the neural tube and yolk sac during embryogenesis. This finding is consistent with previous reports of c-kit expression in these organs^25^,^26^. Immunostaining of sectioned *c-kit*^*H2B-tdTomato/+*^ mouse tissues revealed that the c-kit^H2B-tdTomato^-positive cells co-localized with c-kit antibody in the liver, lung and melanocytes (Supplementary Fig. 3). Further support for the sensitivity and fidelity of this reporter is the observation that cells with low c-kit expression detected by antibody exhibited bright H2B-tdTomato fluorescence (Supplementary Fig. 3b,c).

Next, we examined the location of c-kit^+^ cells in the hearts of *c-kit*^*H2B-tdTomato/+*^mice (Fig. 1). Endocardial cells with nuclear tdTomato expression were observed as early as E8.5 and 9.5 (Fig. 1b,c). Starting from E12.5, cells with strong c-kit^H2B-tdTomato^ expression were dispersed throughout the heart, with the highest density in the inner layers of the atrial and ventricular chambers at all embryonic stages tested (Fig. 1d,e). At postnatal day (P) 1, P30, P60 and P120, c-kit^H2B-tdTomato^–expressing cells were consistently detected in all chambers of the heart (Fig. 1f–i). The broad distribution of c-kit^H2B-tdTomato^-positive cells in the heart from embryonic stages to adulthood is inconsistent with previous studies reporting that c-kit^+^ cells represent a small population of CSCs in the mammalian heart^7^,^12^,^13^,^14^,^15^,^27^.

### c-kit^+^ cells do not express Nkx2.5 or cTnT after E13.5

In the initial characterization of cardiac resident c-kit^+^ cells in the adult rat, c-kit^+^ cells were shown to contain a mixed population of cells exhibiting early stages of myogenic differentiation as demonstrated by the active expression of the early cardiac transcription factors Nkx2.5, Gata4 and Mef2c in the nucleus and of sarcomeric proteins in the cytoplasm of these cells^7^,^15^. To determine whether c-kit^H2B-tdTomato^-positive cells express the cardiac progenitor marker Nkx2.5, we crossed *Nkx2.5*^*H2B-GFP/+*^knock-in mice^28^ with *c-kit*^*H2B-tdTomato/+*^mice to obtain compound heterozygotes (*c-kit*^*H2B-tdTomato/+*^*;Nkx2.5*^*H2B-GFP/+*^). H2B–GFP expression in *Nkx2.5*^*H2B-GFP/+*^mice faithfully recapitulates the endogenous Nkx2.5 pattern^28^. We examined cardiac tissues throughout the embryonic (E9.5–18.5) and postnatal (P1–120) stages (Supplementary Fig. 4). All histological sections from E9.5 to 13.5 hearts and more than 30 sections from E14.5 to P120 hearts were inspected (*n*=3 for each stage). However, no c-kit^H2B-tdTomato^ and Nkx2.5^H2B-GFP^ double-positive cells were found (Supplementary Fig. 4b,d–g), except at E12.5, wherein only 11 double-positive cells were detected in the ventricular septum (Supplementary Fig. 4c, ∼0.007% of total Nkx2.5^H2B-GFP^-positive cells).

To determine whether any c-kit^+^ cells produce sarcomeric or myocardial proteins^7^,^15^, we applied a *cTnT*^*H2B-GFP/+*^ knock-in mouse model with insertion of an *H2B–GFP* cassette into the start codon of *cTnT* (*Tnnt2;* Supplementary Fig. 5a). On examining heart sections from *c-kit*^*H2B-tdTomato/+*^;*cTnT*^*H2B-GFP/+*^ compound heterozygous animals at embryonic and postnatal stages (E8.5–P120), we did not detect any cells in which both markers were co-localized (Supplementary Fig. 5), with the exception of E13.5, where an average of 15 double-positive cells were found within the ventricular septum (Supplementary Fig. 5d, ∼0.009% of total cTnT^H2B-GFP^-positive cells). These observations reveal that c-kit^+^ cells in *c-kit*^*H2B-tdTomato/+*^mice very rarely co-express either Nkx2.5 or cTnT in the embryonic heart and do not co-express these markers in foetal or adult hearts.

### Cardiac c-kit^+^ cells are a subpopulation of endothelial cells

**Figure 2:**
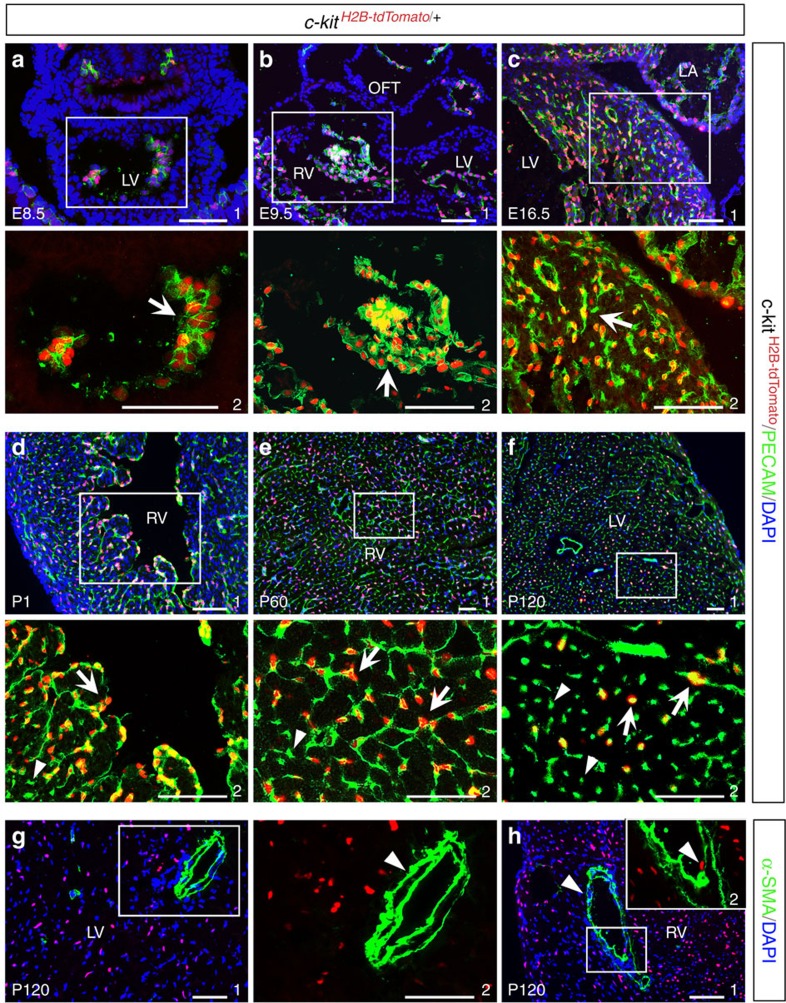
Cardiac c-kit^H2B-tdTomato^ cells are PECAM^+^ endothelial cells. (**a**,**b**) At E8.5 and E9.5, c-kit^H2B-tdTomato^ cells are endocardial (PECAM^+^). (**c**–**f**) c-kit^H2B-tdTomato^ cells express PECAM at E16.5 (**c**) and at P1–120 (**d**–**f**). Arrows indicate PECAM^+^ and tdTomato^+^ double-positive cells. Arrowheads indicate PECAM^+^ and tdTomato^−^ cells. (**g**,**h**) Cardiac smooth muscle cells (α-SMA^+^) are tdTomato^−^ at P120 (arrowheads). a2–h2 are high-magnification images of the areas outlined in a1–h1 (without DAPI), respectively. *n*=3 for each stage. Scale bar, 100 μm.

To further determine the identity of c-kit^+^ cells, we performed immunostaining with antibodies against the endothelial marker PECAM (CD31) and the smooth muscle marker, α-SMA. Surprisingly, at all the stages examined (E8.5–P120), c-kit^H2B-tdTomato^-positive cells were PECAM^*+*^(Fig. 2a-f) but α-SMA^−^ (Fig. 2g,h). This finding suggests that cardiac c-kit^H2B-tdTomato^-positive cells are endothelial cells. Quantitative flow cytometric analysis of 4-month-old hearts demonstrated that ∼43% PECAM^*+*^ cells in the ventricles were also c-kit^+^ (Supplementary Fig. 6). Thus, our results indicate that c-kit^H2B-tdTomato^-positive cells represent a subset of cardiac endothelial cells.

**Figure 3:**
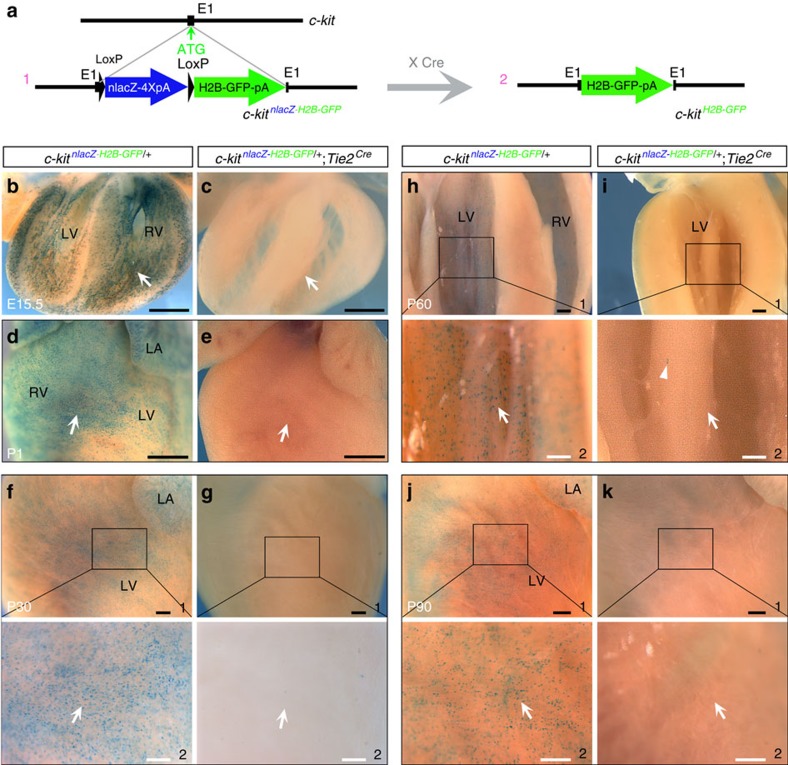
c-kit^nlacZ^ cells are of a Tie2 endothelial lineage. (**a**) Diagram of the *c-kit*^*nlacZ-H2B-GFP/+*^reporter allele (a1). The *c-kit*^*H2B-GFP/+*^ allele is generated when the *nlacZ* cassette is removed by Cre excision (a2). (**b**–**k**) X-gal staining of *c-kit*^*nlacZ-H2B-GFP*/+^ and *c-kit*^*nlacZ-H2B-GFP/+*^;*Tie2*^*Cre*^ littermate hearts at E15.5 (**b**,**c**, sections) and at P1–90 (**d**–**k**). Arrows indicate comparable regions to X-gal^+^ or X-gal^−^ staining. Arrowheads indicate rare X-gal^+^ cells on *c-kit*^*nlacZ-H2B-GFP/+*^;*Tie2*^*Cre*^ hearts, suggesting that most c-kit^+^ cells lose the *nlacZ* gene because they are in the *Tie2*^*Cre*^ lineage. f2–k2 are high-magnification images of the areas outlined in f1–k1, respectively. *n*=3–5 for each stage. Scale bar, 400 μm (black) and 200 μm (white).

tdTomato is a bright fluorescent protein^29^,^30^. We were concerned that the long stability of tdTomato could complicate the detection of transient c-kit expression. To confirm the identity of c-kit^+^ cells identified by *c-kit*^*H2B-tdTomato/+*^, we generated another reporter line, *c-kit*^*nlacZ-H2B-GFP/+*^, by inserting a *LoxP-nlacZ-4XPolyA-LoxP-H2B–GFP* cassette into the *c-kit* start codon (Fig. 3a and Supplementary Fig. 7). H2B–GFP is not detected in this line unless the *nlacZ-4XPolyA* stop cassette is removed by Cre-mediated recombination. We performed whole-mount X-gal staining on *c-kit*^*nlacZ-H2B-GFP/+*^ embryos and found that the c-kit^nlacZ^ signal was not only reliably recapitulated by c-kit mRNA expression, but also consistent with the H2B–tdTomato expression patterns in *c-kit*^*H2B-tdTomato/+*^mice (Supplementary Fig. 2). Furthermore, X-gal staining of whole-mount and sectioned hearts at E15.5–P90 readily detected a broad distribution of c-kit^nlacZ^-positive cells throughout the heart (Fig. 3b,d,f,h, and j), including the endocardium (Fig. 3b,h), similar to the pattern observed in *c-kit*^*H2B-tdTomato/+*^mice. X-gal staining of compound heterozygous littermate hearts bearing an endothelial-specific *Tie2-Cre* allele (*c-kit*^*nlacZ-H2B-GFP/+*^*;Tie2*^*Cre*^) could not detect *c-kit*^*nlacZ*^-positive cells (Fig. 3c,e,g,i and k; less than 10 randomly distributed c-kit^nlacZ^-positive cells were found in the adult heart, representing ∼0.0002% of total c-kit^+^ cells). Consistent with the endothelial nature of c-kit^+^ cells in the heart, c-kit^H2B-GFP^-positive cells generated by Tie2^Cre^ excision were all co-stained with anti-PECAM antibody (Supplementary Fig. 8). Thus, the *c-kit*^*nlacZ-H2B-GFP/+*^ reporter line confirms the endothelial identity of cardiac c-kit^+^ cells.

**Figure 4:**
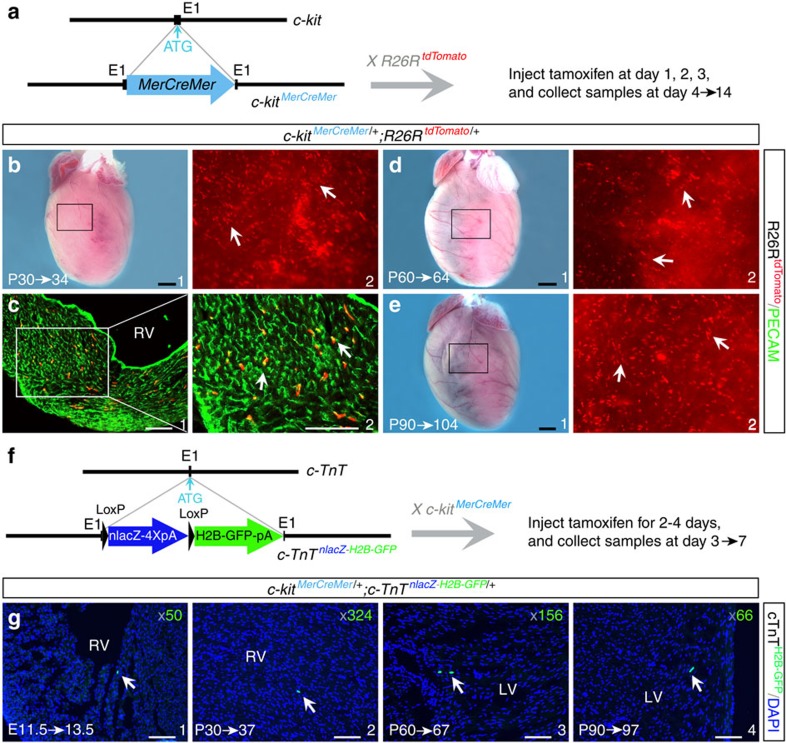
Active c-kit endothelial expression and myogenic potential assayed by transient induction of Cre activity in *c-kit*^*MerCreMer/+*^ mice. (**a**) Diagram of the *c-kit*^*MerCreMer/+*^ allele*. c-kit*^*MerCreMer/+*^ animals were crossed to the *ROSA26R*^*tdTomato*^ reporter line to obtain *c-kit*^*MerCreMer/+*^;*ROSA26R*^*tdTomato/+*^. (**b**–**e**) Cre activity was transiently induced in *c-kit*^*MerCreMer/+*^;*ROSA26R*^*tdTomato/+*^ animals at P30, P60 and P90 by tamoxifen injection on days 1–3. Hearts were harvested on days 4 and 14. Many tdTomato^*+*^ cells (arrows in b2, d2 and e2) were detected in hearts at P34 (b1), P64 (d1) and P104 (e1). These tdTomato^*+*^ cells were PECAM^+^ (c2, arrows, P30→34). b2, d2 and e2 are high-magnification florescent images of the areas outlined in b1, d1 and e1 (bright field), respectively. (**f**) Diagram of the *cTnT*^*nlacZ-H2B-GFP/+*^allele and lineage tracing using *c-kit*^*MerCreMer/+*^;*cTnT*^*nlacZ-H2B-GFP/+*^mice. Cre activity was transiently induced by tamoxifen injection for 4 days on days 1, 2, 3 and 5 (days 1 and 2 for E11.5). Samples were collected on day 7 (day 3 for E11.5). (**g**) cTnT^H2B-GFP^ cells were detected at E13.5, P37, P67 and P97 (arrows), with the total number in the whole heart noted at the upper right corner. Scale bar, 1 mm (black) and 100 μm (white).

To further address the issue of stability of both H2B–tdTomato and nlacZ proteins, we analysed cardiac c-kit cells with the third reporter allele *c-kit*^*MerCreMer/+*^, in which an inducible *MerCreMer* cassette was inserted into the *c-kit* start codon (Fig. 4a and Supplementary Fig. 9). *c-kit*^*MerCreMer/+*^;*ROSA26R*^*tdTomato/+*^mice were subsequently generated by crossing with *ROSA26R*^*tdTomato/+*^ mice. In the absence of tamoxifen treatment, no tdTomato-expressing cells were detected in the adult hearts. To confirm whether c-kit is actively expressed in the postnatal heart, we injected tamoxifen at P30, P60 or P90 for 3 consecutive days (days 1, 2 and 3), and immediately collected cardiac tissues for analysis at day 4 (P30→34, P60→64) or 14 (P90→104). This treatment consistently resulted in tdTomato labelling of a large number of cells in the heart (Fig. 4b,d,e) that also expressed PECAM (Fig. 4c). This result further confirms that cardiac c-kit^+^ cells are endothelial (Figs 2 and 3), and supports the previous observation that cardiac c-kit^+^ cell progeny are endothelial^19^.

### c-kit^+^ endothelial cells are identified by immunostaining

*c-kit*^*H2B-tdTomato/+*^, *c-kit*^*nlacZ-H2B-GFP/+*^ and *c-kit*^*MerCreMer/+*^ animals are heterozygous null for *c-kit* (*c-kit*^*+/−*^). Haploinsufficiency of c-kit could affect *c-kit* regulation *in vivo*^20^,^31^,^32^,^33^, possibly leading to ectopic cardiac expression. To determine whether ectopic *c-kit* expression occurs in the reporter mouse hearts, we performed immunostaining at embryonic (E11.5–15.5) and postnatal stages (P1–60) using c-kit antibody on mice of four different genotypes: wild type, *c-kit*^*H2B-tdTomato/+*^ (*c-kit*^*+/−*^), *c-kit*^*H2B-tdTomato/MerCreMer*^(*c-kit*^*−/−*^) and *c-kit*^*MerCreMer/MerCreMer*^(*c-kit*^*−/−*^). Using c-kit antibody, we frequently detected cells in wild-type hearts that were dually labelled with c-kit and PECAM (Supplementary Fig. 10a4,d4,g2 and Supplementary Fig. 11a,f,h,i). In *c-kit*^*H2B-tdTomato/+*^ animals, c-kit antibody immunoreactivity co-localized with c-kit^H2B-tdTomato^ (Supplementary Fig. 10b2, e2,h2 and Supplementary Fig. 11b,c), although the immunofluorescence was decreased compared with that in wild-type animals. Reduced c-kit immunoreactivity in *c-kit*^*H2B-tdTomato/+*^ tissues is consistent with the *c-kit*^*+/−*^ genetic background (theoretically 50% c-kit protein reduction in *c-kit*^*+/−*^). Importantly, c-kit antibody staining was completely undetectable in *c-kit*^*−/−*^mutant hearts or lungs, even with Tyramide Signal Amplification (TSA) amplification (Supplementary Figs 10c,f and 11d,e), demonstrating the specificity of the antibody staining. Therefore, immunostaining with c-kit antibody also reveals that cardiac c-kit^+^ cells are endothelial and indicates that no ectopic cardiac c-kit expression occurs in the new knock-in mouse models employed.

### Resident c-kit^+^ cells rarely differentiate into cardiomyocytes

To further determine the myogenic potential of c-kit^+^ cells during heart formation, we applied *cTnT*^*nlacZ-H2B-GFP/+*^ cardiomyocyte-specific reporter mice with the *LoxP-nlacZ-4XPolyA-LoxP-H2B-GFP* cassette targeted into *cTnT* start codon. cTnT^H2B-GFP^ expression is detected in cardiomyocytes when Cre is expressed in the myocardium or myogenic precursor cells (Fig. 4f). We crossed *c-kit*^*MerCreMer/+*^ mice with *cTnT*^*nlacZ-H2B-GFP/+*^mice and injected tamoxifen in *c-kit*^*MerCreMer/+*^;*cTnT*^*nlacZ-H2B-GFP/+*^ animals. After two doses of tamoxifen administration (days 1 and 2) to pregnant mice (E11.5 embryos) or four doses (days 1, 2, 3 and 5) to P30, P60 and P90 mice, we collected hearts for analysis at E13.5 or at P37, P67 and P97, respectively. All cardiac sections were assessed for cTnT^H2B-GFP^-positive cells. On average, approximately 50, 324, 156 and 66 cells were found in hearts (*n*=3 for each group) at E13.5, P37, P67 and P97, respectively (Fig. 4g), representing <0.04% of cardiomyocytes at corresponding stages (<0.007% after P90). This finding demonstrates that the myogenic potential of c-kit^+^ cells, if any, is extremely low in both embryonic and postnatal hearts.

**Figure 5:**
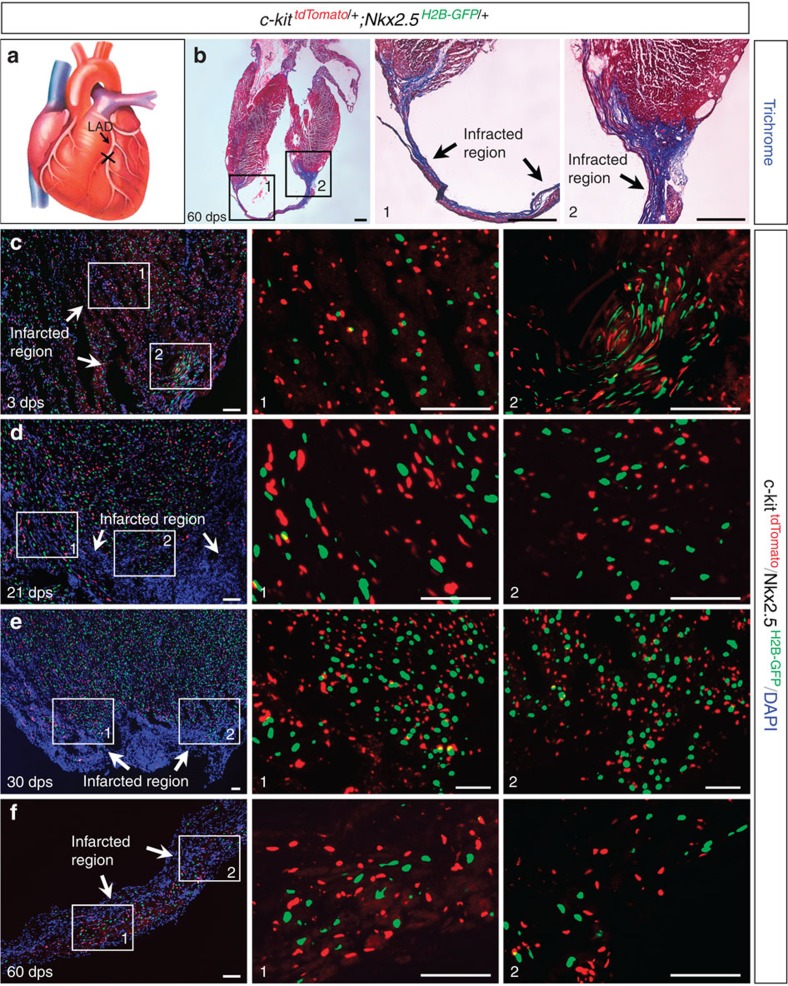
c-kit^+^ cells do not co-express Nkx2.5 in the injured region. (**a**) Diagram of LAD ligation. (**b**) Masson trichrome staining shows the infarcted region of a *c-kit*^*H2B-tdTomato/+*^;*Nkx2.5*^*H2B-GFP/+*^heart at 60 days post-surgery (dps). b1 and b2 are high-magnification images of the numbered outlined areas in **b**. (**c**–**f**) No c-kit^H2B-tdTomato^/Nkx2.5^H2B-GFP^ double-positive cells were found in the infarcted regions at 3 (**c**), 21 (**d**), 30 (**e**) and 60 dps (**f**). c1/c2, d1/d2, e1/e2, and f1/f2 are high-magnification images of the numbered outlined areas in **c, d, e** and **f**, respectively. Scale bar, 500 μm (black) and 50 μm (white).

**Figure 6:**
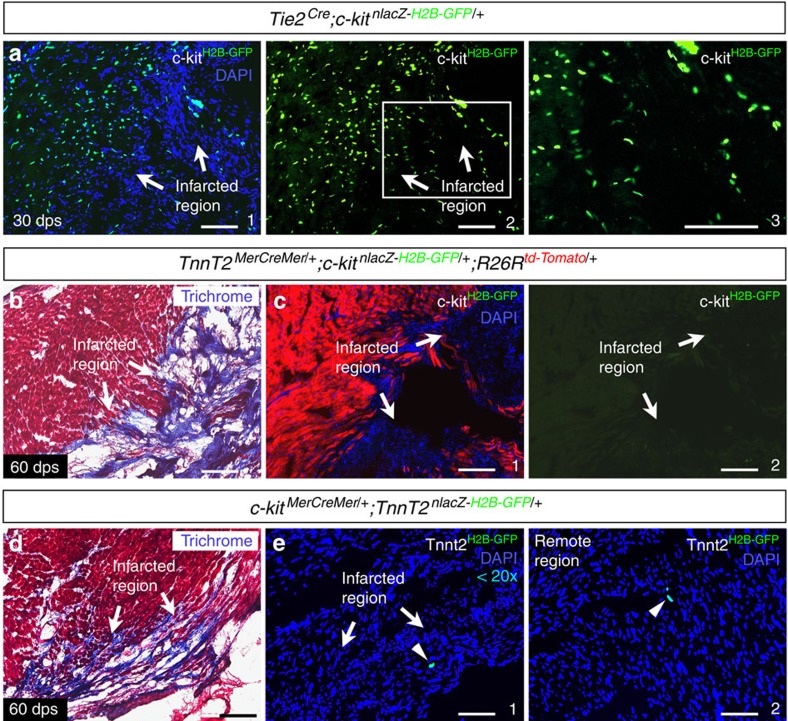
Cell type and lineage of c-kit^+^ cells in the injured heart. (**a**) c-kit^H2B-GFP^-positive cells were present in the infarcted region of *Tie2*^*Cre*^;*c-kit*^*nlacZ-H2B-GFP/+*^ hearts at 30 dps. a2 is green channel of a1, and a3 is high-magnification image of the area outlined in a2. (**b**) Masson trichrome staining of *cTnT*^*MerCreMer/+*^;*c-kit*^*nlacZ-H2B-GFP/+*^;*ROSA26R*^*tdTomato/+*^ hearts at 60 dps shows the infarcted region. (**c**) Adjacent section of **b**. ROSA26R^tdTomato^ signal indicates myocardial cells after tamoxifen induction (c1). No c-kit^H2B-GFP^ cells were observed in the infarcted zone (arrows). c2 is green channel of c1. (**d**) Masson trichrome staining of *c-kit*^*MerCreMer/+*^;*cTnT*^*nlacZ-H2B-GFP/+*^ hearts at 60 dps. (**e**) Adjacent section of d shows a few cTnT^H2B-GFP^ cells (<20) that were found in the infarcted zone (e1, arrowhead). cTnT^H2B-GFP^ cells were also present in a remote, uninjured region (e2, arrowhead). Scale bar, 100 μm.

Previous studies have reported that within 4 weeks of myocardial infarction in adult mouse hearts, the number of c-kit/Nkx2.5 double-positive myogenic precursors significantly increased in the injured region, and some of these myogenic precursors transformed into proliferative cardiomyocytes^7^,^15^. To directly investigate the differentiation potential of cardiac c-kit^+^ cells post myocardial infarction, we ligated the left anterior descending (LAD) coronary artery of *c-kit*^*H2B-tdTomato/+*^;*Nkx2.5*^*H2B-GFP/+*^ mice (2–5 months old, *n*=12, Fig. 5a,b). Examination of cardiac sections at 1, 3, 7, 21, 30 and 60 days post-surgery (dps) revealed many c-kit^H2B-tdTomato^-positive cells in the infarcted region (Fig. 5c–f). However, no c-kit^H2B-tdTomato^ and Nkx2.5^H2B-GFP^ double-positive cells were found in the injured area at any stage tested (Fig. 5c1–f1). To further determine the cell identity of these c-kit^+^ cells, we performed LAD ligation on *Tie2*^*Cre*^;*c-kit*^*nlacZ-H2B-GFP/+*^ mice (2–4 months old, *n*=3). c-kit^H2B-GFP^-positive cells were readily detected in the infarcted region, demonstrating that they retained their endothelial nature after injury (Fig. 6a).

A recent study reported that a subpopulation of endothelial cells yields progeny with CSC characteristics in the adult mouse heart^34^. This subpopulation purportedly arises from endothelial–mesenchymal transition and gives rise to cardiomyocytes that contribute to heart renewal^34^. To determine whether c-kit^+^ endothelial cells produce CSCs that further differentiate into cardiomyocytes following cardiac injury, we performed LAD ligation on *cTnT*^*MerCreMer/+*^;*c-kit*^*nlacZ-H2B-GFP/+*^;*ROSA26R*^*tdTomato/+*^ mice (2–4 months old, *n*=4, Fig. 6b). *cTnT*^*MerCreMer/+*^ mediates specific and effective myocardial recombination after tamoxifen induction^35^. If *c-kit*^*nlacZ-H2B-GFP/+*^ cells become cardiomyocytes and if c-kit expression is maintained in these cells, then c-kit^H2B-GFP^-positive cells would be detected. However, after tamoxifen was injected at 3–7 dps and 31–35 dps (three tamoxifen treatments for each period), we detected no c-kit^H2B-GFP^-positive cells in the infarcted region (Fig. 6c), although myocardial recombination was widely detected in and adjacent to the infarcted region (as revealed by ROSA26R^tdTomato^ staining, Fig. 6c). Furthermore, examination of adult *c-kit*^*MerCreMer/+*^;*cTnT*^*nlacZ-H2B-GFP/+*^ mice after LAD ligation (3–5 months old, *n*=3, Fig. 6d) revealed <20 cTnT^H2B-GFP^-positive cells per heart (∼0.002% of total myocardial cells) throughout the injured region (Fig. 6e). cTnT^H2B-GFP^-positive cells could also be detected in remote uninjured regions (∼30 cells, ∼0.003% of total myocardial cells, Fig. 6e), suggesting that the cTnT^H2B-GFP^-positive cells found in the injured region are likely not a response to cardiac injury. These cardiac injury mouse models revealed that the myocardial potential of c-kit^+^ endothelial cells, if any, is extremely low. However, these data do not preclude the possibility that c-kit^−^ cardiac endothelial cells may have the potential for endothelial–mesenchymal transition and myocardial differentiation.

### A rare population of cardiac c-kit^+^ cells are cardiomyocytes

In the lineage tracing experiments used to determine the myocardial potential of c-kit^+^ cells during development and after cardiac injury in *c-kit*^*MerCreMer/+*^;*cTnT*^*nlacZ-H2B-GFP/+*^ animal models, very small number of cTnT^H2B-GFP^-positive cells was detected (Fig. 4g, ∼66–156 cells; and Fig. 6e, ∼20 cells). In all cases, the number was extremely low when compared with the total number of c-kit^H2B-tdTomato^-positive cells (<0.005%) or myocardial cells (<0.015%) in whole hearts. The origin of these rare cells is unknown. These cells may be derived from uncommitted cells originally expressing c-kit, or they could be cardiomyocytes that express both c-kit and cTnT due to a rare stochastic event. To explore these possibilities, we examined *cTnT*^*MerCreMer/+*^;*c-kit*^*nlacZ-H2B-GFP/+*^ adult mouse hearts (2–4 months old, uninjured) after tamoxifen injection for 2 consecutive days (days 1 and 2). At days 3, 7 and 30, we detected ∼20–30 c-kit^H2B-GFP^-positive cells per heart after examining all the heart sections (*n*=3, Supplementary Fig. 12). This result suggests that a very small number of resident c-kit cells are cardiomyocytes (∼0.005% of total c-kit^+^ cells and ∼0.002% of total myocardial cells in the heart). Notably, the number of c-kit^H2B-GFP^-positive cells detected in *cTnT*^*MerCreMer/+*^;*c-kit*^*nlacZ-H2B-GFP/+*^ hearts (∼20–30, Supplementary Fig. 12) is less than the number of cTnT^H2B-GFP^-positive cells in *c-kit*^*MerCreMer/+*^;*cTnT*^*nlacZ-H2B-GFP/+*^ hearts (∼66–156, Fig. 4g3). This is probably due to much higher levels of *cTnT* expression than *c-kit* expression and/or to differential sensitivity of the reporters to Cre-mediated recombination.

## Discussion

Currently, the identity and differentiation potential of resident c-kit^+^ cells in the mammalian heart are the central questions in cardiac regenerative medicine. For more than a decade, cardiac c-kit^+^ cells have been described as a multipotent cell population with a regenerative capacity^7^,^12^,^13^,^14^,^15^,^27^. If c-kit expression defines a ‘stem' or ‘undifferentiated' state, then c-kit should not maintain to be expressed in any differentiated cardiac cell type such as the endothelium or myocardium. However, our studies of three informative reporter alleles in mice (*c-kit*^*H2B-tdTomato/+*^, *c-kit*^*nlacZ-H2B-GFP/+*^ and *c-kit*^*MerCreMer/+*^;*ROSA26R*^*tdTomato/+*^) combined with c-kit immunostaining consistently revealed that c-kit actively labels an endothelial population in mouse hearts during development and into adulthood. These results argue against the current paradigm that c-kit is a marker of CSCs or that cardiac c-kit^+^ cells are uncommitted^7^,^13^,^14^.

A recent c-kit lineage tracing study by van Berlo *et al.*^19^ revealed that c-kit^+^ cells rarely became cardiomyocytes, instead entering into an endothelial cell fate. Our study supports this observation. However, van Berlo's study leaves the possibility that c-kit may label cardiac stem or progenitor cells that possess endothelial potential. Concerns were also raised regarding the fidelity and sensitivity of the mouse models employed in van Berlo's study^20^. Here with a new set of mouse models, we demonstrated the endothelial nature of cardiac resident c-kit^+^ cells. Our observations explain the c-kit endothelial lineage findings by van Berlo *et al.*, and also explain the low myocardial potential of these cells (because they are in fact endothelial cells). Active c-kit expression in the committed endothelium during heart formation indicates that c-kit is not an appropriate marker of resident CSCs, including CSCs destined for an endothelial fate. Our studies of myocardial infarction injury mouse models suggest that c-kit^+^ endothelial cells rarely (if ever) de-differentiate into CSCs to contribute to myocardial repair. Future studies are warranted to determine the mechanisms by which c-kit^+^ cells contribute to heart repair (if any) based on their endothelial identity.

## Methods

### Mouse models

*c-kit*^*H2B-tdTomato/+*^, *c-kit*^*nlacZ-H2B-GFP/+*^ and *c-kit*^*MerCreMer/+*^ knock-in mouse models were generated by inserting *LoxP-4XPloyA-LoxP-H2B-tdTomato-FRT-Neo-FRT, LoxP-nlacZ-4XPloyA-LoxP-H2B-GFP-FRT-Neo-FRT and MerCreMer-FRT-Neo-FRT* cassettes, respectively, into the start codon of the *c-kit* locus (with disruption of endogenous ATG) through homologous recombination in 129/SvJ ES cells. In the targeting constructs, the insertion cassettes are flanked by 3.7 kb 5′ and 3.8 kb 3′ homologous arms (Supplementary Figs 1,7,9). The targeting vectors were linearized and electroporated individually in mouse ES cells. ES cells were screened by long-range PCR (Roche, Cat. 04829069001) with two pairs of primers (P1+P2 and P3+P4, Supplementary Figs 1,7,9). The sequences of the PCR fragments from the positive ES cells were further verified by DNA sequencing. The male chimeric mice carrying the targeted cassette in their germ line were crossed with Black Swiss females to generate F1 heterozygous mice. The Neo cassette flanked by two FRT sites was removed by crossing F1 mice with Flippase deleter mice^36^. *c-kit*^*LoxP-4XPloyA-LoxP-H2B-tdTomato/+*^ (*c-kit*^*STOP-H2B-tdTomato/+*^) mice were crossed with Protamine-Cre^37^ to remove the *4XPloyA* stop cassette and to obtain *c-kit*^*H2B-tdTomato/+*^. The P1–4 sequences are: P1, 5′-GGGTCTTCCTATATCTCCCTAGCT-3′; P2 (*c-kit*^*STOP-H2B-tdTomato/+*^), 5′-CCAAATAAGCTTGGATCCGGAACC-3′; P2 (*c-kit*^*nlacZ-H2B-GFP/+*^), 5′-ATTCGCGTCTGGCCTTCCTGTAGC-3′; P2 (*c-kit*^*MerCreMer/+*^), 5′-CTCTTCTTCTTGGGCATGGTCTGC-3′; P3, 5′-TACCTGCCCATTCGACCACCAAGC-3′; and P4, 5′-ACCTCACACAGAACCTCCAGCAAT-3′.

*Nkx2.5*^*H2B-GFP/+*^, *cTnT*^*MerCreMer/+*^ and *ROSA26R*^*tdTomato/+*^ (*R26R*^*tdTomato/+*^) mouse lines were previously described^28^,^35^,^38^. For *cTnT*^*nlacZ-H2B-GFP/+*^ mouse line, a *LoxP-nlacZ-4XPloyA-LoxP-H2B-GFP* cassette was targeted to the *cTnT* start codon (manuscript was submitted). The *cTnT*^*H2B-GFP/+*^ mouse was obtained by crossing *cTnT*^*LoxP-nlacZ-4XPloyA-LoxP-H2B-GFP/+*^ (*cTnT*^*nlacZ-H2B-GFP/+*^) mice with *Protamine-Cre* mice^37^. *Nkx2.5*^*H2B-GFP/+*^ and *cTnT*^*H2B-GFP/+*^ mice were crossed with *c-kit*^*H2B-tdTomato/+*^ to obtain *c-kit*^*H2B-tdTomato/+*^;*Nkx2.5*^*H2B-GFP/+*^ and *c-kit*^*H2B-tdTomato/+*^;*cTnT*^*H2B-GFP/+*^ compound heterozygous mice. The compound heterozygous mice had normal heart development.

Tamoxifen (Sigma, Cat. T5648) was intraperitoneally injected into mice (0.12 mg g^−1^ body weight). Genomic DNA was prepared from yolk sacs or tails for genotyping. Mouse husbandry was conducted in accordance with an approved protocol by Icahn School of Medicine at Mount Sinai Institutional Animal Care and Use Committee (IACUC) and was in compliance with institutional and governmental regulations (PHS Animal Welfare Assurance A3111-01).

### X-gal staining

For whole-mount staining, the tissues were fixed in 4% paraformaldehyde for 30 min on ice. After the tissues were quickly washed twice in PBS, they were stained in X-gal solution (5 mM potassium ferricyanide, 5 mM potassium ferrocyanide, 2 mM MgCl_2_, and 1 mg ml^−1^ X-gal) overnight at room temperature. For section staining, the heart tissues were fixed in 4% paraformaldehyde for 30 min, washed with PBS, soaked in 30% sucrose overnight and then embedded in optimal cutting temperature compound (Tissue-Tek). Coronal sections of hearts were prepared using a cryostat. The sections were re-fixed in 4% paraformaldehyde for 5–7 min followed by staining with X-gal solution at 37 °C overnight. At least three mice from each stage were examined.

### RNA *in situ* hybridization

Whole-mount RNA *in situ* hybridization of mouse embryos was performed using Wilkinson's protocol^39^.

### Immunofluorescence

Mouse tissues were fixed in 4% paraformaldehyde for 30 min, washed with PBS, soaked in 30% sucrose overnight and then embedded in optimal cutting temperature. Cryosections of heart (coronal) were cut to 8 μm thickness. The primary antibodies used in this study were rat anti-PECAM (CD31; 1:100, BD Biosciences, Cat. 553371), goat anti-c-kit (CD117; 1:20 to 1:40 for postnatal hearts and 1:40 to 1:100 for embryonic hearts, R&D systems, AF1356) and mouse anti-α-SMA (1:100, Sigma, Cat. A5228). Alexa Fluor 488- or 594-conjugated secondary antibodies (1:500; Invitrogen) were applied to detect the corresponding primary antibodies. A TSA kit (Perkin Elmer, Cat. NEL741001KT) was applied to amplify fluorescent signals resulting from c-kit antibody staining. Horseradish peroxidase–conjugated anti-goat IgG (1:500; Abcam, Cat. ab97110) was used as a secondary antibody when TSA was applied to enhance immunostaining.

### Flow cytometry

Mouse ventricular endothelial cells were obtained by enzymatic dissociation of the heart following standard perfusion procedures^40^ with modifications. Briefly, adult mice (4 months old) were injected with heparin 20 min before heart excision and anaesthetized by isoflurane inhalation. Hearts were quickly removed from the chest and perfused with Ca^2+^-free solution containing collagenase type II (Worthington, Lakewood, NJ, USA). Ventricles were cut into small pieces and gently minced with a Pasteur pipette. Dissociated cells were transferred to a 50 ml Falcon tube and kept in Tyrode's solution at room temperature for 5–10 min. Ventricular cardiomyocytes settled on the bottom of the tube. Most non-cardiomyocyte cells were then collected without disturbing the cardiomyocyte layer for flow cytometric analysis.

The cells were washed in PBS with 0.5% bovine serum albumin (BSA). The cell suspension was adjusted to a concentration of 1 × 10^6^ cells ml^−1^, and single cells were incubated in blocking buffer (PBS with Fc blocking IgG and 1% BSA) for 30 min at room temperature. PECAM/CD31-APC–conjugated antibody (BD, Cat. 561814) was added to the blocking buffer (5 μl per 10^6^ cells). The cells were incubated with gentle shaking for 30 min at room temperature in the dark. Red blood cell lysis buffer was added, and then the samples were incubated at room temperature for 10 min to eliminate red blood cells. The cells were subsequently washed twice and then resuspended in PBS with 0.5% BSA for flow cytometry (Beckman Coulter MoFlo Cytomation).

### Myocardial infarction

Myocardial infarction was induced by LAD coronary artery ligation in mice of both genders with body weights ranging from 25 to 34 g (2–6 months old)^41^. Briefly, mice were anaesthetised intraperitoneally with ketamine (0.065 mg g^−1^ body weight), acepromazine (0.001 mg g^−1^ body weight) and xylazine (0.013 mg g^−1^ body weight). After thoracotomy, LAD ligation was performed with a 7-0 silk suture 3–4 mm from the tip of the left auricle. The successful performance of LAD ligation was verified by visual inspection of the colour of the apex. The chest was closed with a 6-0 silk suture, and the skin was closed with 4-0 silk sutures. All mice were housed under identical conditions and were given water and food *ad libitum*.

### Cell counting

Specific genotype mice were applied to count the number of c-kit^+^ (*c-kit*^*H2B-tdTomato/+*^), Nkx2.5^+^ (*Nkx2.5*^*H2B-GFP/+*^) and cTnT^+^ (*cTnT*^*H2B-GFP/+*^) cells in the hearts. Cryosections (10 μm, coronal) were cut through the heart. For embryonic stages, every fifth section was collected. For hearts older than P30, 1 in every 20 sections was collected. Cells from five representative sections were counted both manually and automatically using ImageJ software with images acquired on a fluorescence microscope. By comparing the numbers acquired by manual counting and ImageJ automatic counting, the thresholds of particle size and intensity were set in ImageJ. Cells from the remaining sections were counted by ImageJ with the same threshold. The total cells were calculated by adding the cell numbers for all sections. The number of cardiomyocytes in the adult heart was divided by 2 considering that 85–90% of these cells are binucleated in mice^42^. As a result, ∼1.5-1.7 × 10^5^ Nkx2.5^+^ or cTnT^+^ cells were calculated in E12.5–13.5 mouse hearts, ∼1.05 × 10^6^ myocardial cells were calculated in the adult mouse hearts, and ∼2.1–4.2 × 10^6^ c-kit^+^ were calculated in the adult mouse hearts (P30–P90).

## Additional information

**How to cite this article:** Sultana, N. *et al.* Resident c-kit^+^ cells in the heart are not cardiac stem cells. *Nat. Commun.* 6:8701 doi: 10.1038/ncomms9701 (2015).

## Supplementary Material

Supplementary Information Supplementary Figures 1-12
