# Supplementary material for: Resident c-kit+ cells in the heart are not cardiac stem cells
Source: Nat Commun. 2015 Oct 30;6:8701. doi: 10.1038/ncomms9701 (PMC4846318; doi:10.1038/ncomms9701)
Supplement: Supplementary Information — Supplementary Figures 1-12 [file ncomms9701-s1.pdf]

Supplementary Figures

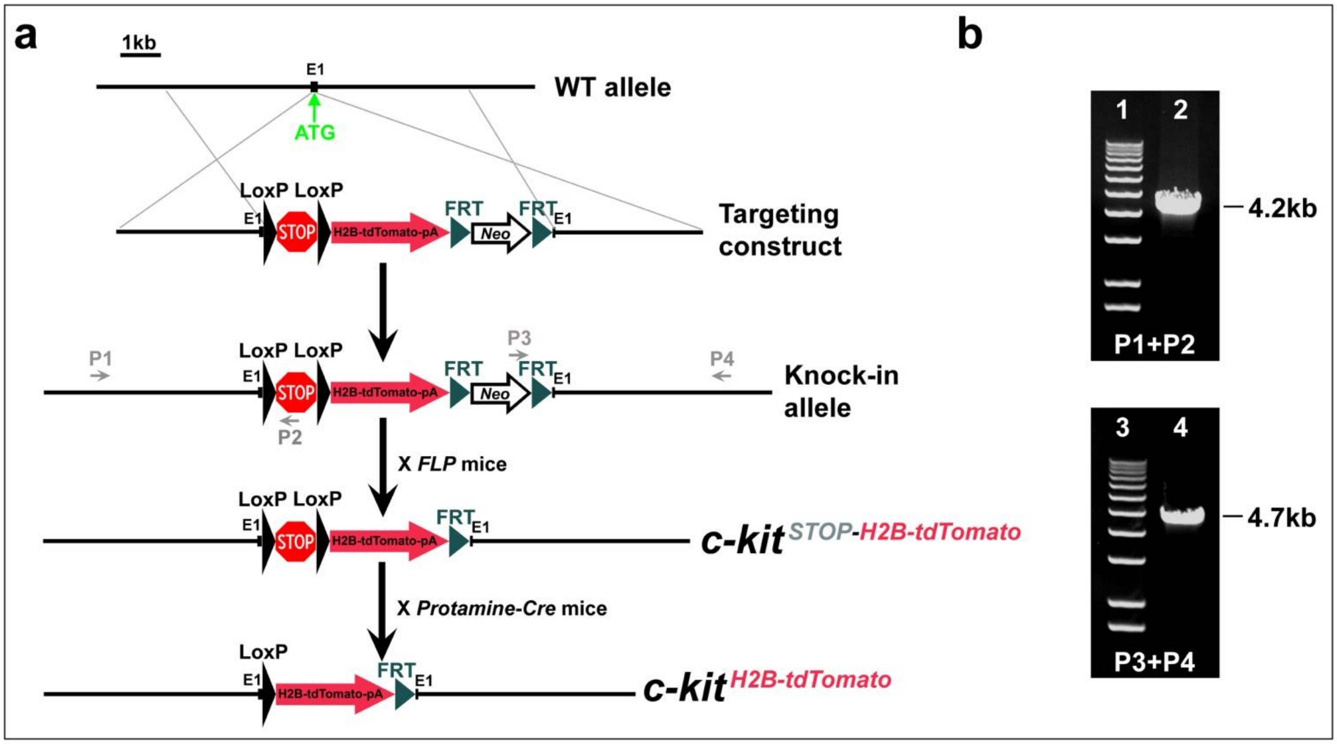

Supplementary Figure 1. Generation of *c-kit*$^{H2B\text{-}tdTomato/+}$ knock-in mice. (a) Schematic representation of the targeting strategy. The targeting construct contains a 3.7 kb 5' homologous arm and a 3.8 kb 3' homologous arm. The *LoxP-4XPolyA-LoxP-H2B-tdTomato-FRT-Neo-FRT* cassette was inserted in the ATG locus of *c-kit*. *c-kit*$^{4XPolyA\text{-}tdTomato\text{-}Neo/+}$ (*c-kit*$^{STOP\text{-}tdTomato\text{-}Neo/+}$) mice were generated from targeted ES cells and were crossed to Flippase deleter mice to remove the *Neo* cassette. Protamine-Cre mice were further crossed to *c-kit*$^{STOP\text{-}H2B\text{-}tdTomato/+}$ mice to generate *c-kit*$^{H2B\text{-}tdTomato/+}$ knock-in mice. **(b)** PCR analysis of genomic DNA from the targeted ES cells. Two fragments (4.2-kb/5' arm and 4.7-kb/3' arm) were amplified by long-range PCR using primers P1 (external to 5' arm) and P2 (in *STOP* cassette), P3 (in *Neo* cassette) and P4 (external to 3' arm), respectively.

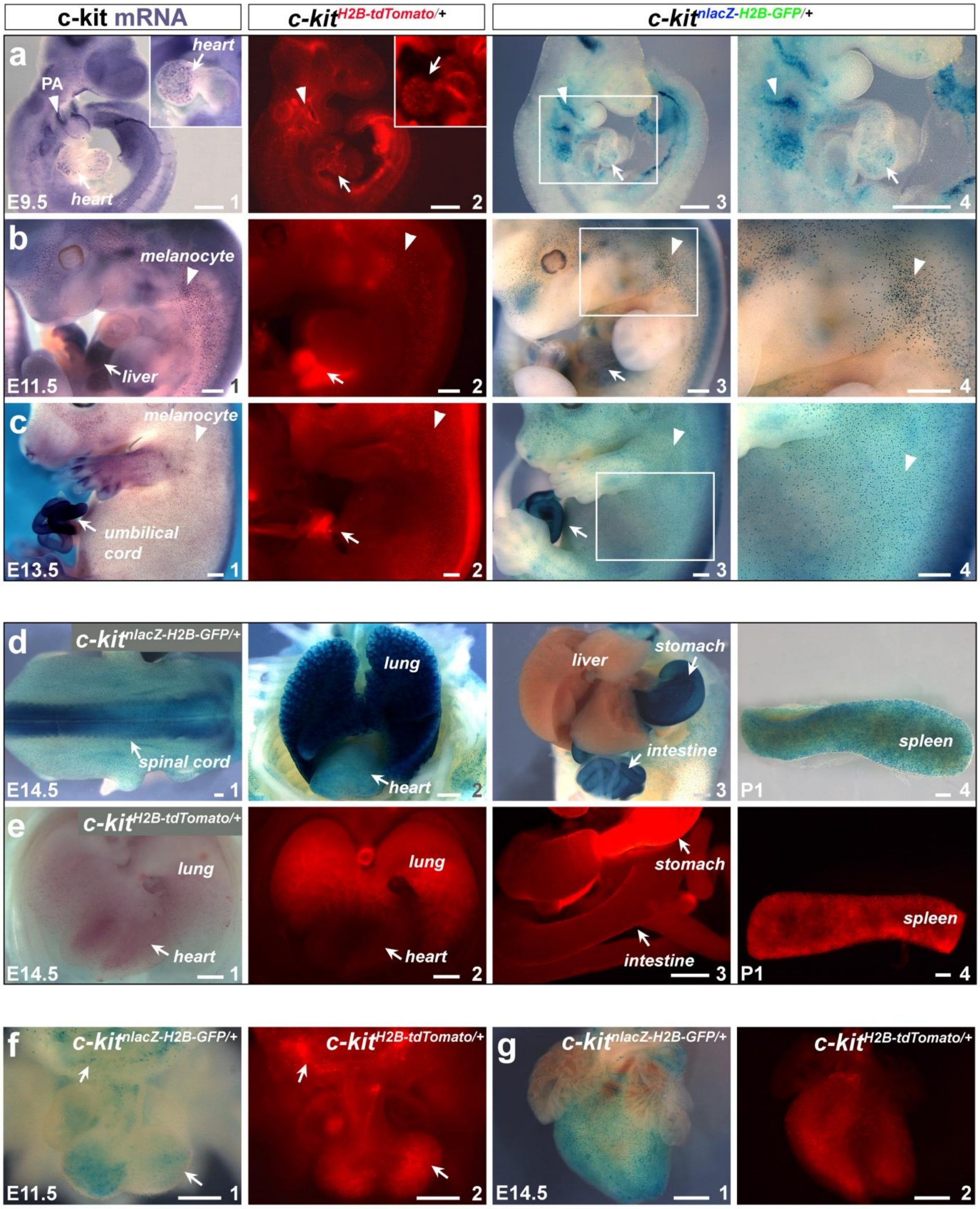

Supplementary Figure 2. c-kit$^{H2B\text{-}tdTomato}$ and c-kit^{nlacZ} recapitulate endogenous c-kit expression. (a-c) c-kit expression was examined by whole-mount RNA *in situ* hybridization (a1, b1, and c1), c-kit$^{H2B\text{-}tdTomato}$ (a2, b2, and c2) and c-kit^{nlacZ} (a3, b3, and c3) at E9.5 (a), E11.5 (b) and E13.5 (c), respectively. a4, b4, and c4 are high-magnification images of the areas outlined in a3, b3, and c3. The inserted images in a1 and a2 show high-magnification images of the heart. Arrows and arrowheads indicate concordant *c-kit* expression in the pharyngeal arches and heart (E9.5), liver (E11.5), melanocytes (E11.5, E13.5) and umbilical cord (E13.5). **(d-g)** Comparable c-kit$^{H2B\text{-}tdTomato}$ and c-kit^{nlacZ} expression is observed in the lung (d2 and e2, E14.5), stomach and intestine (d3 and e3, E14.5), spleen (d4 and e4, P1) and hearts (f and g, E11.5 and E14.5). c-kit^{nlacZ} is also detected in the neural tube and liver (d1 and d3, data not shown for *c-kit$^{H2B\text{-}tdTomato}$*). e1 is a bright image for e2. Cardiac c-kit^{nlacZ} and c-kit$^{H2B\text{-}tdTomato}$ expression is significantly lower than in the lung (d2 and e2). n>3 for each stage. Scale bar, 400 μm.

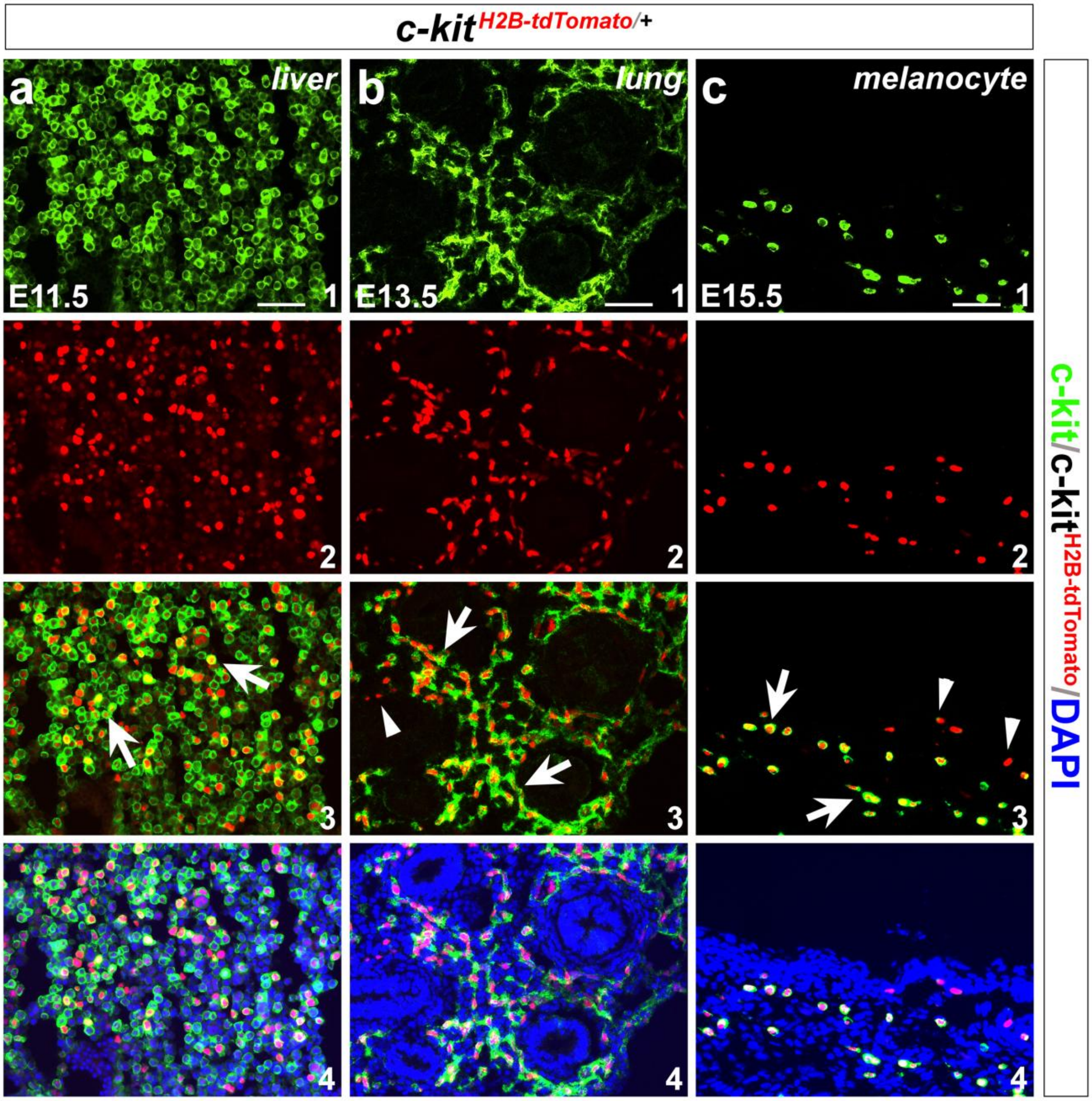

Supplementary Figure 3. c-kit$^{H2B\text{-}tdTomato}$ recapitulates endogenous c-kit expression with enhanced sensitivity. Immunostaining of c-kit in the liver (a1, E11.5), lung (b1, E13.5) and melanocytes (c1, E15.5). a2, b2, and c2 show c-kit$^{H2B\text{-}tdTomato}$ expression. a3, b3, and c3 are merged images of 1 and 2. a4, b4, and c4 are merged images of 1 and 2 with DAPI. Arrows in a3, b3, and c3 indicate co-localization of c-kit$^{H2B\text{-}tdTomato}$ with c-kit antibody. Arrowheads indicate c-kit$^{H2B\text{-}tdTomato}$ staining despite a very low c-kit antibody signal. *n*=5 for each stage. Scale bar, 50 μm.

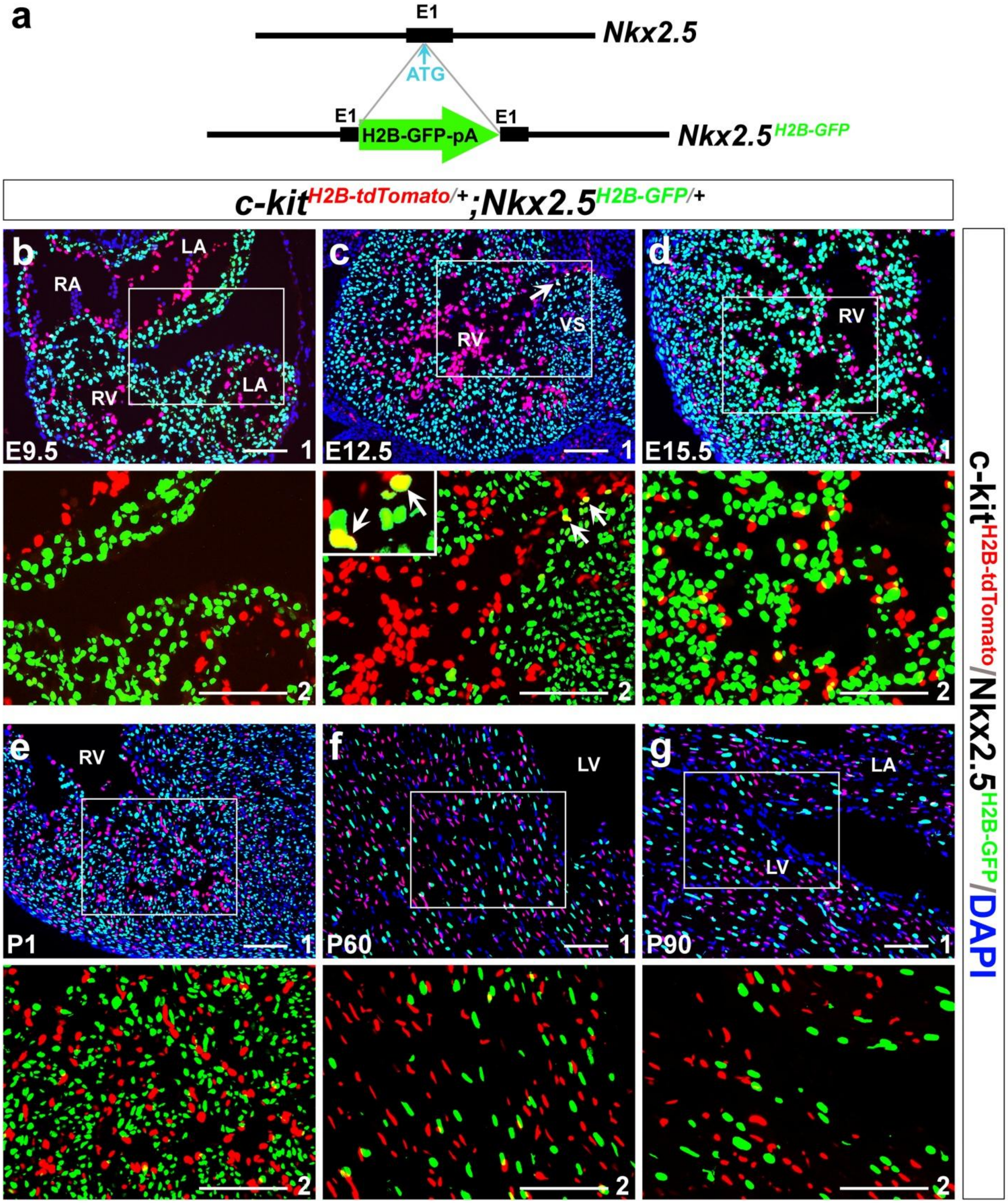

Supplementary Figure 4. Nkx2.5 and c-kit rarely co-localize in mouse hearts. (a) Diagram of the *Nkx2.5*$^{H2B\text{-}GFP/+}$ mouse. **(b-g)** *c-kit*$^{H2B\text{-}tdTomato/+}$*;Nkx2.5*$^{H2B\text{-}GFP/+}$ heart sections were examined. No double–positive cells were found in E9.5 (b2), E15.5 (d2), and P1-90 (e2, f2, and g2) hearts. Inserted image in c2 shows high magnification of c1 in a region indicated by an arrow in the ventricular septum, with a few Nkx2.5$^{H2B\text{-}GFP}$/c-kit$^{H2B\text{-}tdTomato}$ double–positive cells (arrows in c2 inserted image). b2-g2 (without DAPI) are high-magnification images of the areas outlined in b1-g1 (with DAPI), respectively. *n*=3 for each stage. Scale bar, 100 μm.

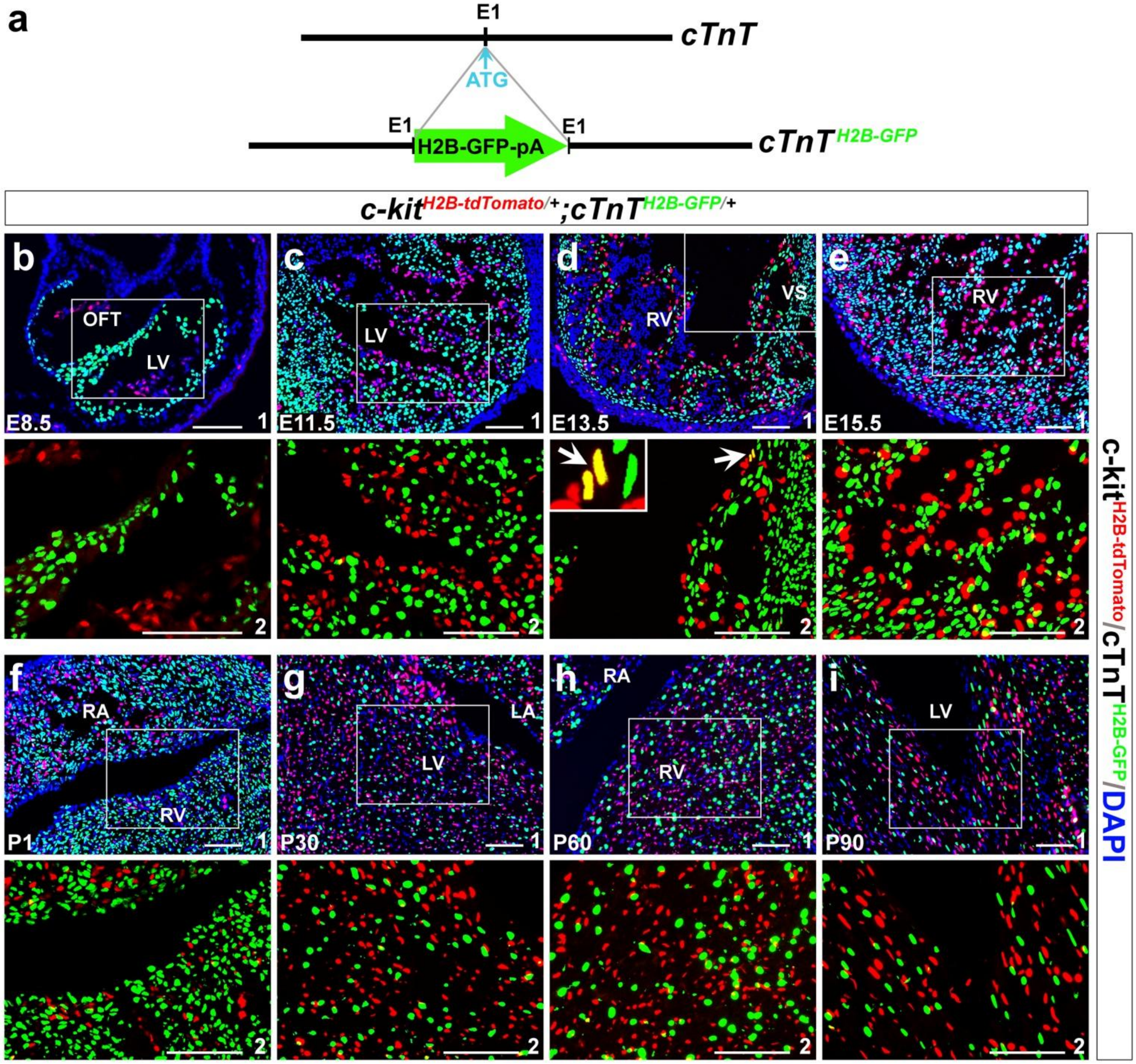

Supplementary Figure 5. Rare cTnT and c-kit double–positive cells in mouse hearts. (a) Diagram of the *cTnT*$^{H2B\text{-}GFP/+}$ mouse. **(b-i)** *c-kit*$^{H2B\text{-}tdTomato/+}$*;cTnT*$^{H2B\text{-}GFP/+}$ heart sections were examined. No double–positive cells were found in E8.5-15.5 (b2, c2, and e2) and P1-90 (f2, g2, h2, and i2) hearts. In d2, the inserted image shows higher magnification in the region labeled by an arrow in the ventricular septum, with a few cTnT$^{H2B\text{-}GFP}$/c-kit$^{H2B\text{-}tdTomato}$ double–positive cells (arrows in the inserted image). b2-i2 are high-magnification images (without DAPI) of areas outlined in b1-i1 (with DAPI), respectively. *n*=3 for each stage. Scale bar, 100 μm.

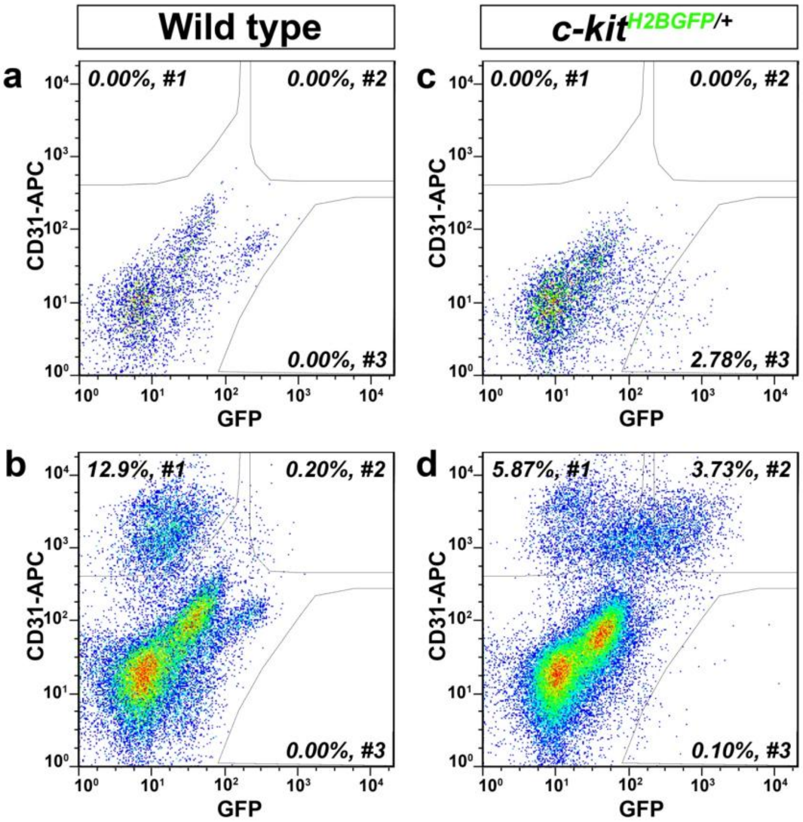

Supplementary Figure 6. Representative flow cytometric staining profiles of PECAM/CD31 expression in the ventricular endothelial cells from wild-type and *c-kit*$^{\mathit{H2B\text{-}GFP/+}}$ mice. *c-kit*$^{H2B\text{-}GFP/+}$ mice were obtained by crossing *c-kit*$^{nlacZ\text{-}H2B\text{-}GFP/+}$ mice to *Protamine-Cre* mice (**Supplementary Figure 7**). **(a)** Control wild-type cardiac cells without CD31-APC antibody, **(b)** CD31–positive cells in the wild-type mouse heart, **(c)** *c-kit*$^{H2B\text{-}GFP/+}$ cells without CD31-APC antibody, and **(d)** CD31–positive cells in the *c-kit*$^{H2B\text{-}GFP/+}$ mouse heart. Group 1: CD31-APC-positive cells; Group 2: CD31-APC and GFP double–positive cells; Group 3: GFP-positive cells. Flow cytometric analysis revealed that ~43% (39-46%) endothelial cells from the *c-kit*$^{H2B\text{-}GFP/+}$ mouse hearts (4 months old) are GFP$^+$. *n*=3 for each group.

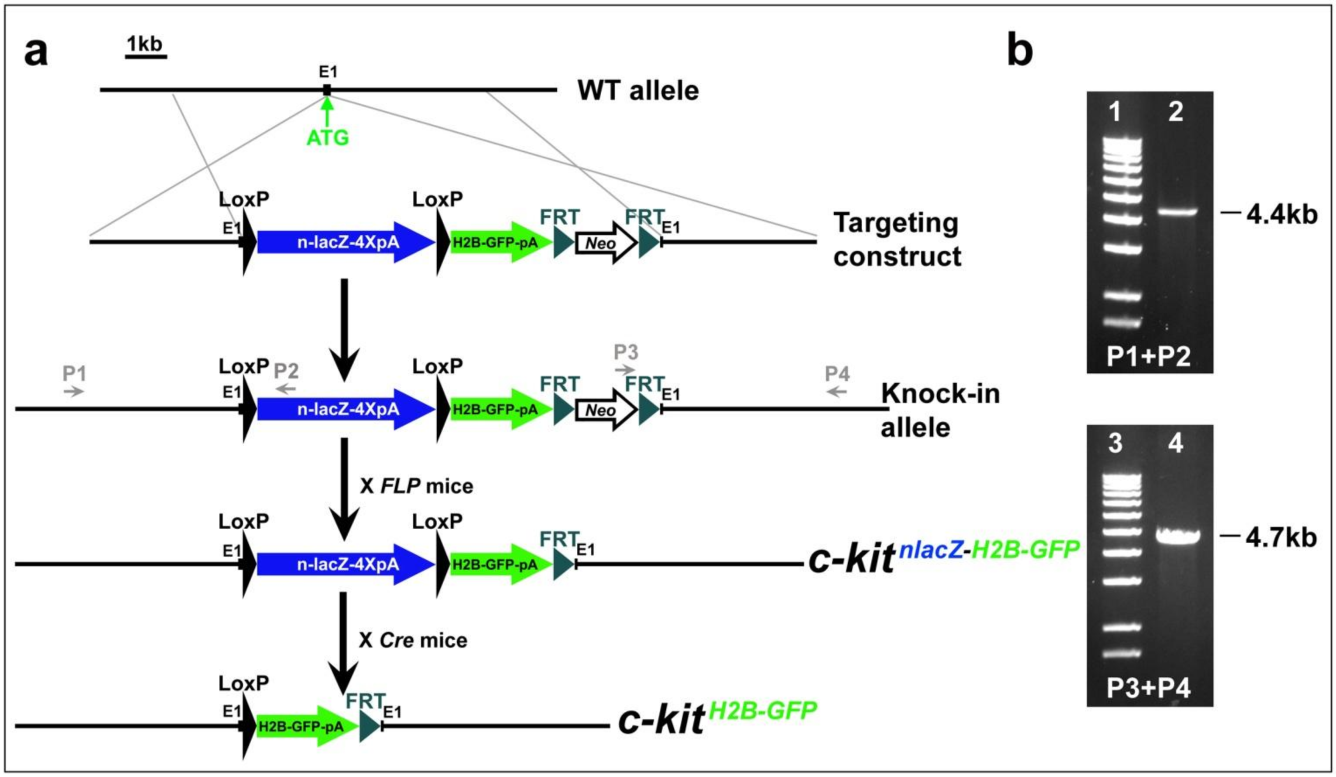

Supplementary Figure 7. Generation of *c-kit*$^{nlacZ\text{-}H2B\text{-}GFP/+}$ mice. (a) Schematic representation of the targeting strategy. The targeting vector contains a 3.7 kb 5' homologous arm and a 3.8 kb 3' homologous arm. The *LoxP-nlacZ-4XPolyA-LoxP-H2B-GFP-FRT-Neo-FRT* cassette was inserted in the ATG locus of *c-kit*. *c-kit*$^{nlacZ\text{-}H2B\text{-}GFP\text{-}Neo/+}$ mice derived from the positive ES cells were crossed to Flippase deleter mice to obtain *c-kit*$^{nlacZ\text{-}H2B\text{-}GFP/+}$ animals. *c-kit*$^{H2B\text{-}GFP/+}$ mice were generated by mating Cre mice to *c-kit*$^{nlacZ\text{-}H2B\text{-}GFP/+}$ mice. **(b)** Two fragments (4.4-kb/5' arm and 4.7-kb/3' arm) were amplified by long range PCR using primers P1 (external to the 5' arm) and P2 (in the *nlacZ* cassette), P3 (in the *Neo* cassette) and P4 (external to the 3' arm), respectively.

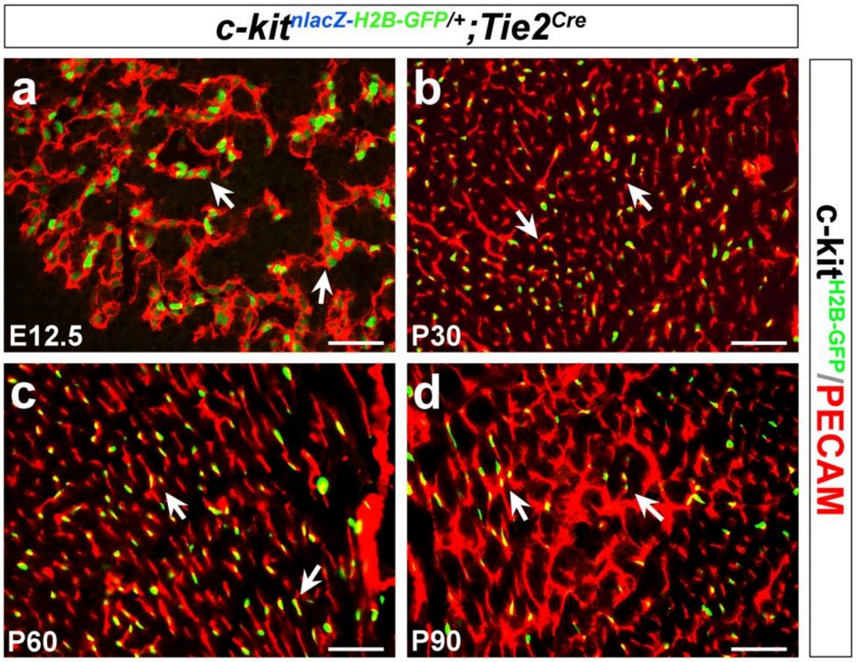

Supplementary Figure 8. c-kit$^{H2B-GFP}$ cells generated by *Tie2*^{Cre} excision in *c-kit*$^{nlacZ-H2B-GFP/+}$;*Tie2*^{Cre} hearts are PECAM^{+} endothelial cells at E12.5 (a), P30 (b), P60 (c) and P90 (d). Scale bar, 50 μm.

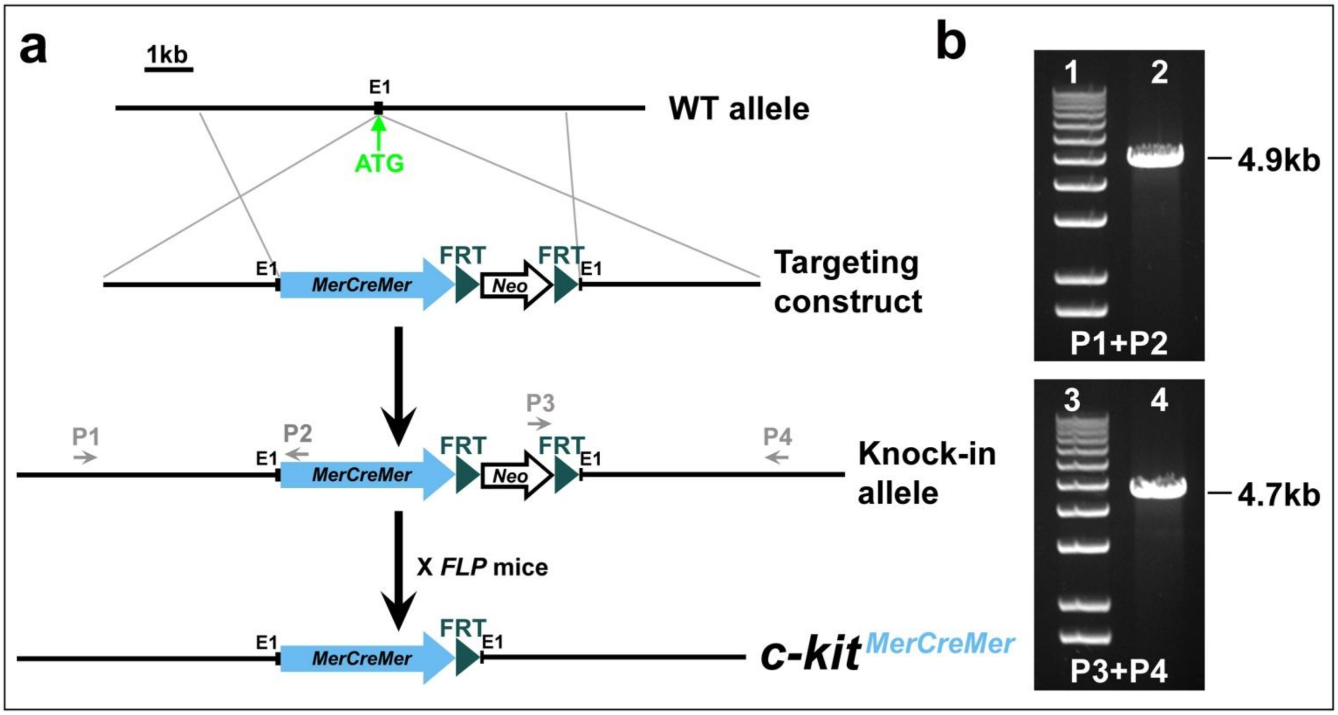

Supplementary Figure 9. Generation of *c-kit*$^{MerCreMer/+}$ knock-in mice. (a) Schematic representation of the targeting strategy. The targeting construct contains a 3.7 kb 5' homologous arm and a 3.8 kb 3' homologous arm. The *MerCreMer-FRT-Neo-FRT* cassette was inserted in the ATG locus of *c-kit*. *c-kit*$^{MerCreMer\text{-}Neo/+}$ mice derived from the targeted ES cells were crossed to Flippase deleter mice to remove the *Neo* cassette. **(b)** Two fragments (4.9-kb/5' arm and 4.7-kb/3' arm) were amplified by long-range PCR using primers P1 (external to the 5' arm) and P2 (in the *MerCreMer* cassette), P3 (in the *Neo* cassette) and P4 (external to the 3' arm), respectively.

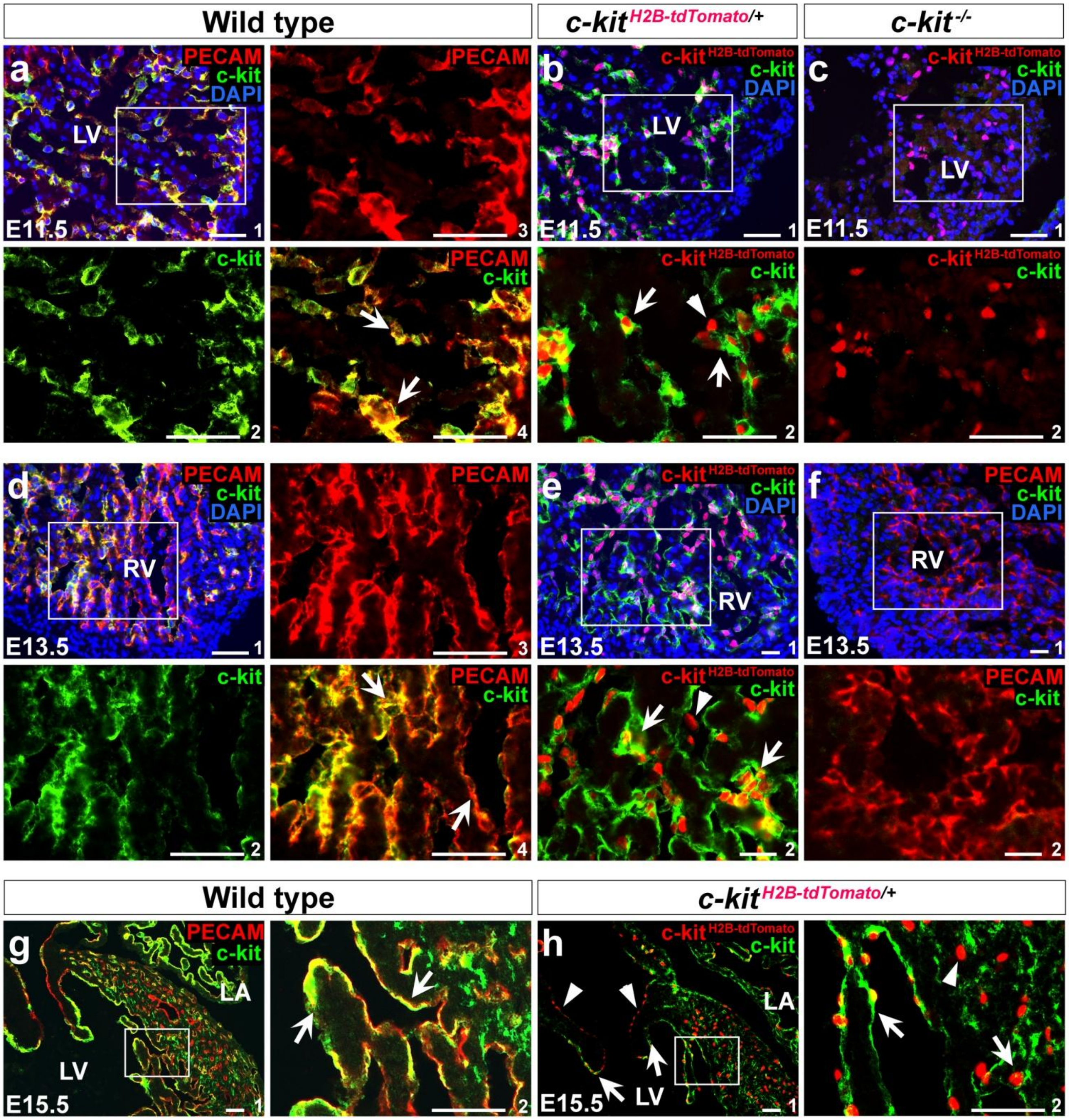

Supplementary Figure 10. Embryonic c-kit expression in wild-type, heterozygous and mutant hearts as determined by c-kit antibody immunostaining. (a, d, g) In the wild-type, c-kit is fully co-localized with endothelial marker PECAM at E11.5 (a), E13.5 (d) and E15.5 (g). a1, d1, and g1 are heart sections in the ventricular chambers. a2-4, d2-4, and g2 are high-magnification images for a1, d1, and g1 in the outlined areas. a2/d2 and a3/d3 are c-kit and PECAM immunostaining, respectively, and a4 and d4 are merged images of a2/a3 and d2/d3, respectively. a4, d4, and g2 show co-localization of c-kit and PECAM staining in the endocardium (arrows). **(b, e, h)** In *c-kit*$^{H2B\text{-}tdTomato/+}$ hearts, c-kit$^{\text{H2B-tdTomato}}$ signals are primarily co-localized with PECAM (b2, e2, and h2, arrows). A few c-kit$^{\text{H2B-tdTomato}}$ cells exhibit very low c-kit staining levels (b2, e2, and h2, arrowheads). b2, e2, and h2 are high-magnification images of the areas outlined in b1, e1, and h1, respectively. **(c, f)** In *c-kit*$^{-/-}$ hearts (c, *c-kit*$^{H2B\text{-}tdTomato/MerCreMer}$; and f, *c-kit*$^{MerCreMer/MerCreMer}$), c-kit antibody staining cannot be detected, although c-kit$^{\text{H2B-tdTomato}}$ (c) and PECAM (f) expression is unaffected. b2, c2, e2, f2, and h2 are high-magnification images of the areas outlined in b1, c1, e1, f1, and h1, respectively. *n*=3-5 for each stage. Scale bar, 50 μm.

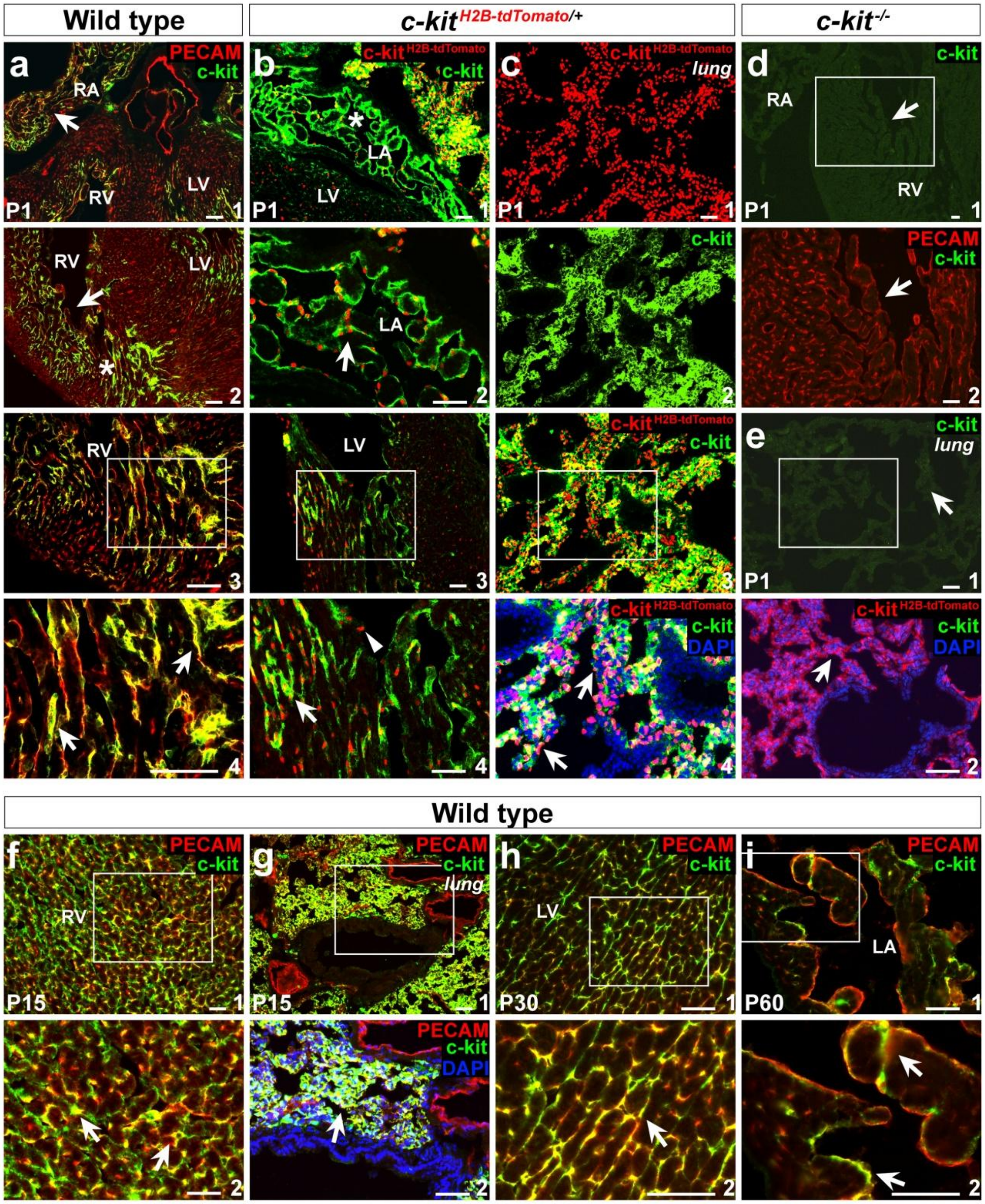

Supplementary Figure 11. Postnatal c-kit expression in the wild-type, heterozygous and mutant hearts and lungs determined by c-kit antibody immunostaining. (a) c-kit$^+$ cells (detected by c-kit antibody) are present in atria (a1, arrow) and ventricles (a2, arrow) of wide type heart at P1. a3 is a high magnification image for a2 in region labeled by asterisk. c-kit$^+$ cells express PECAM (arrows, a4). **(b)** In *c-kit*$^{H2B\text{-}tdTomato/+}$ hearts, c-kit$^+$ cells are detected in the atria (b1 and b2, arrows) and ventricles (b3 and b4), and they are mostly c-kit$^{H2B\text{-}tdTomato}$–positive cells. b2 is a high magnification image for b1 in region marked by asterisk. Arrowhead indicates c-kit$^{H2B\text{-}tdTomato}$–positive cells with low c-kit antibody staining (b4). **(c)** As control, c-kit$^{H2B\text{-}tdTomato}$–positive cells in lung are mostly c-kit$^+$ at P1 in *c-kit*$^{H2B\text{-}tdTomato/+}$. **(d,e)** In *c-kit*$^{-/-}$ heart (d, *c-kit*$^{MerCreMer/MerCreMer}$) and lung (e, *c-kit*$^{H2B\text{-}tdTomato/MerCreMer}$), c-kit antibody staining is absent, although PECAM (d2) and c-kit$^{H2B\text{-}tdTomato}$ (e2) expression appears unaffected. **(f-i)** c-kit expression is co-localized with PECAM on P15 (f), P30 (h) and P60 (i) hearts (wild-type). g shows co-localized PECAM and c-kit staining in lung at P15. a4, b4, c4, d2, e2, f2, g2, h2, and i2 are high magnification images in areas outlined in a3, b3, c3, d1, e1, f1, g1, h1, and i1, respectively. *n*=3-5 for each stage. Scale bar, 50 μm.

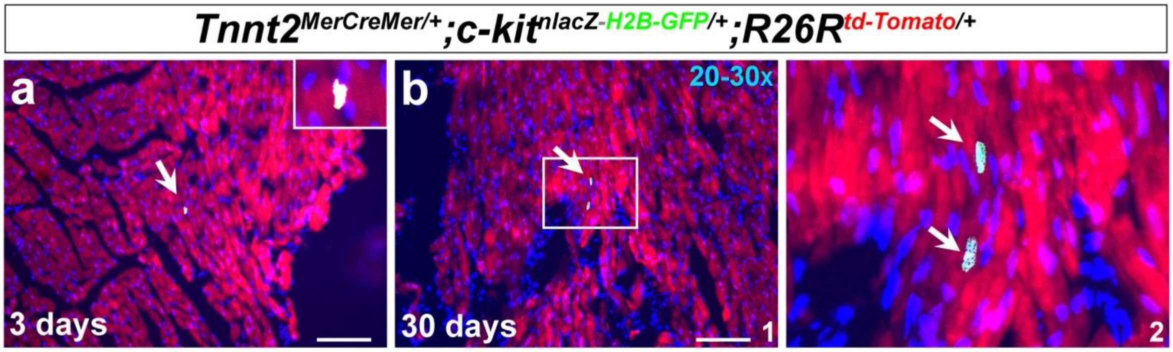

Supplementary Figure 12. A very small number of c-kit^{+} cells are cardiomyocytes. Approximately 20-30 c-kit$^{H2B\text{-}GFP}$ cells were found (arrows) on the adult *cTnT$^{MerCreMer/+}$;c-kit$^{nlacZ\text{-}H2B\text{-}GFP/+}$;ROSA26R$^{tdTomato/+}$* hearts at 3 (a) and 30 days (b) after tamoxifen induction. The upright corner image in a is a high magnification in the area with the arrow. b2 is a high magnification image of b1 in the area outlined. Arrows indicate c-kit$^{H2B\text{-}GFP}$–positive cells. Scale bar, 100 μm.
